# A case of COVID-19 with an ultralong incubation period

**DOI:** 10.1017/ice.2020.221

**Published:** 2020-05-11

**Authors:** Yujin Wang, Qingwen Wang, Kai Wang, Congkuan Song, ZiXin Guo, Weidong Hu

**Affiliations:** 1 Department of Thoracic Surgery, ZhongNan Hospital of Wuhan University, Wuhan, Hubei, China; 2 Hubei Key Laboratory of Tumor Biological Behaviors, Hubei Cancer Clinical Study Center, Hubei, China; 3 Township government of Shanpo, Jiangxia District, Wuhan, Hubei, China

*To the Editor—*A large global outbreak of coronavirus disease 2019 (COVID-19), caused by SARS-CoV-2, has become a critical public health issue since December 2019.^1,2^ SARS-CoV-2 spreads by human-to-human transmission mainly via droplets or direct contact, and it has been estimated to have mean incubation period of 6.4 days and a basic reproduction number of 2.24–3.58.^3,4^ An understanding of the incubation period is essential to detect epidemiological cases and helpful to determine the quarantine and medical observation period of intimate contacts. Whether SARS-CoV-2 can be transmitted during the incubation period remains controversial.^5-7^ Here, we report the case of a patient who had a long incubation period (38 days) and infected 1 close contact with SARS-CoV-2 during the incubation period.

A 50-year-old man (person 1), who worked in Hankou District, Wuhan, China, returned home on January 21, 2020, to a small village in the rural area of Wuhan City. First, he took the city subway, then he boarded an intercity train to a rural town, and finally his brother-in-law (person 2) drove him home by car. After returning home, person 1 lived with his elder brother (person 3), who was a single man with mild deafness who lived alone during ordinary (nonoutbreak) times. From then on, both men did not leave their home. Person 3 lived in an independent house in the small village and had neither gone to other places nor had contacted other people since January 1, 2020. Person 2 sent some food to the house of person 3 on January 17, but at that time, person 3 was not at home because he was doing farm work in the field. Persons 1 and 3 were at home together beginning January 21, 2020, and during this period they met nobody.

Person 3 developed symptoms of cough and wheezing on February 5, 2020. On February 6, he underwent a chest computed tomography (CT) examination and was suspected of COVID-19 pneumonia. Person 3 was admitted to the hospital for treatment on February 7, and the next day his throat swab test for SARS-CoV-2 was positive by quantitative RT-PCR (qRT-PCR) analysis. During the hospitalization, person 3 was in stable condition without severe complications such as acute respiratory distress syndrome or shock. After treatment, person 3 was discharged on February 23 and isolated himself.

Person 1 began a 14-day quarantine on February 7 and ended on February 21. On February 24, he underwent a routine CT examination before returning home. The results showed very slight inflammation of the lung, but at that time, he still did not have any symptoms. On February 27, person 1 started to have a cough, and he underwent another chest CT test, which indicated progressive pulmonary inflammation, so he was admitted to the hospital for quarantine and treatment. His SARS-CoV-2 test was also positive by qRT-PCR analysis. After treatment, he had 2 throat swab test for SARS-CoV-2 a week apart. The results of these tests were negative, and another chest CT image showed that the inflammation was absorbing. Thus, person 1 was discharged on March 2.

**Fig. 1.**
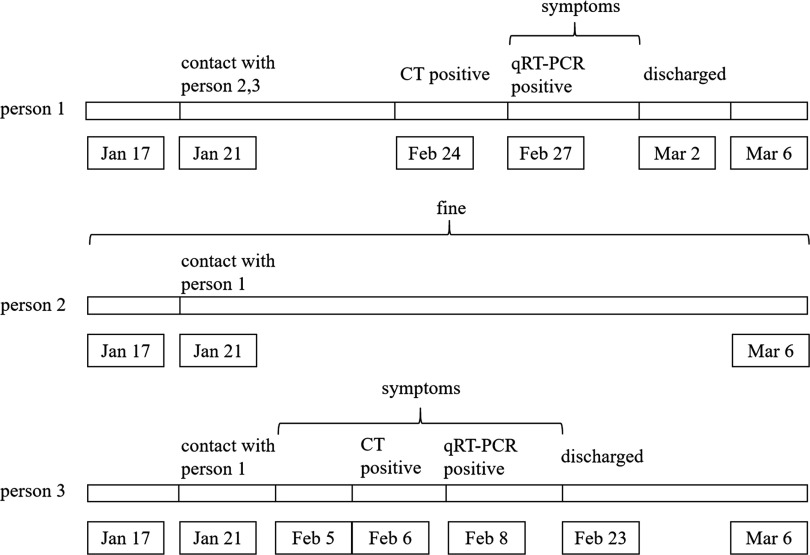
Chronology of contacts, symptom onset and identification of positive SARS-CoV-2 findings on CT and qRT-PCR of 3 people. Note. CT, computed tomography; qRT-PCR, quantitative reverse-transcriptase polymerase chain reaction.

Person 2 was the other man who had initial contact with person 1 on January 21. He lived with his family (his wife and son) from then on, and he had a family dinner with his other 2 relatives (his brother and sister-in-law) on January 24. These 5 people were in good health until March 6. Under strict isolation control, the villagers basically had no contact, and no other people were reported to have COVID-19 in the village where person 1 and person 3 lived (Fig. 1).

Except for person 1, who came from the Hankou District of Wuhan, the epidemic area on January 21, when COVID-19 was rapidly spreading, person 3 had no contact with other people, especially with infected or suspected persons, for more than a month before he was identified of COVID-19 on February 8. Person 3’s infection was suspected to have come from person 1, of whom the incubation period was 16 days.

Based on the exposure history and the onset of symptoms of person 1, we reasonably speculated that the incubation period of person 1 was at least 38 days, which was calculated from January 21 to February 27. By contrast, the incubation period of these 2 patients was much longer than the mean incubation period reported by Backer et al,^3^ and person 1’s case may represent the longest incubation period of all cases reported up to now.^8,9^

The long incubation period and mild symptoms of these 2 patients is notable and may be due to the weak virulence of the SARS-CoV-2 type to which they were exposed. Tang et al^10^ divided SARS-CoV-2 into 2 major types based on population genetic analyses of 103 SARS-CoV-2 genomes, 1 of which was less aggressive. The weak virulence of the virus is a double-edged sword. On one hand, it poses less threat to the infected, and on the other hand, people may carry the virus for a long time without realizing it and therefore transmit it to others inadvertently.

In this report, person 3 was infected by person 1 during the incubation period, which provides evidence that SARS-CoV-2 was contagious during the incubation period. The long incubation period of this COVID-19 case may contribute to our knowledge of SARS-CoV-2 and may imply that a longer observation or isolation period for contacts should be considered in SARS-CoV-2 epidemic areas.
